# Assessment of the neuroprotective effect of green synthesized iron oxide nanoparticles capped with curcumin against a rat model of Parkinson’s disease

**DOI:** 10.22038/IJBMS.2023.73124.15892

**Published:** 2024

**Authors:** Yasser Ashry Khadrawy, Eman Nasr Hosny, Haitham Sharf Eldein Mohamed

**Affiliations:** 1 Medical Physiology Department, Medical Division, National Research Centre, Giza, Egypt; 2 Biophysics Department, Faculty of Science, Cairo University, Giza, Egypt

**Keywords:** FeONPs-Cur, Monoamines, Motor activity, Oxidative stress, Parkinson’s disease

## Abstract

**Objective(s):**

The current study aims to investigate the protective effect of iron oxide nanoparticles capped with curcumin (FeONPs-Cur) against motor impairment and neurochemical changes in a rat model of Parkinson’s disease (PD) induced by reserpine.

**Materials and Methods:**

Rats were grouped into control, PD model induced by reserpine, and PD model treated with FeONPs-Cur (8 rats/group). The open field test was used to assess motor activity. The concentration of dopamine (DA), norepinephrine (NE), serotonin (5-HT), lipid peroxidation (MDA), reduced glutathione (GSH), and nitric oxide (NO), and the activities of Na^+^,K^+^,ATPase, acetylcholinesterase (AchE), and monoamine oxidase (MAO) were determined in the midbrain and striatum. Data were analyzed by ANOVA at *P*-value<0.05.

**Results:**

The PD model exhibited a decrease in motor activity. In the midbrain and striatum of the PD model, DA, NE, and 5-HT levels decreased significantly (*P*-value<0.05). However, an increase in MAO, NO, and MDA was observed. GSH, AchE and Na^+^,K^+^,ATPase decreased significantly in the two brain areas. FeONPs-Cur prevented the decline of dopamine and norepinephrine and reduced oxidative stress in both areas. It also prevented the increased MAO activity in the two areas and the inhibited activity of AchE and Na^+^,K^+^,ATPase in the midbrain. These changes were associated with improvements in motor activity.

**Conclusion:**

The present data indicate that FeONPs-Cur could prevent the motor deficits induced in the PD rat model by restoring dopamine and norepinephrine in the midbrain and striatum. The antioxidant activity of FeONPs-Cur contributed to its protective effect. These effects nominate FeONPs-Cur as an antiparkinsonian candidate.

## Introduction

Parkinson’s disease (PD) is the most common global neurodegenerative motor disorder (1). It impacts 1–2 per thousand people at any given time, and its prevalence rises with age, accounting for 1% of individuals over the age of 60 (2).

PD is manifested when over 50% of dopaminergic neurons are lost in the substantia nigra pars compacta (3). PD is characterized by motor impairments such as resting tremors, bradykinesia, muscular rigidity, and nonmotor disorders including gastrointestinal symptoms, autonomic dysfunction, pain, and sleep disturbances caused by neuronal loss in nondopaminergic areas (4). Although the underlying factors causing dopaminergic neuron death are still poorly understood, oxidative stress has been thought to be closely related to the loss of neurons in PD (5). The relation between oxidative stress and PD pathogenesis has been evidenced by animal models of the disease generated by neurotoxins, including 1-methyl-4-phenyl-1,2,3,6-tetrahydropyridine, 6-hydroxydopamine, and rotenone, which produce reactive oxygen species (ROS) and the progressive death of dopaminergic neurons (6, 7). Even though almost all therapies focus on elevating striatal dopamine levels, current research showed that the cholinergic system participates in information processing in the striatum and may become a potential target for antiparkinsonian drugs (8). Although several efficient medications were produced to treat PD, their inability to penetrate the blood-brain barrier makes it difficult to administer them (9). PD is increasingly considered a multisystem neurodegeneration influencing several neurotransmission systems (10).

As the precise cause of PD has not been determined, therapeutic discoveries are still in progress. There is no known cure for the disease, so treatments attempt to manage the symptoms rather than slow or prevent the disease’s progression. Disease management varies from drugs, surgeries, therapy, or a combination of different treatments (11). 

Iron oxide (Fe_3_O_4_) NPs represent a class of biocompatible nanomaterials that have been extensively utilized for diagnosis, bioimaging, and therapy (12, 13). They exhibited a neuroprotective effect in a PD model, reducing the accumulation of α-synuclein and activation of caspase-3. More notably, dietary Fe_3_O_4_ NPs diminished ROS in aged Drosophila, increased their ability to climb, and extended their life span (14). On the other hand, iron oxide NPs may cause neurodegeneration due to the accumulation of iron in the cerebral tissue and their ability to induce oxidative stress and protein aggregation. It was reported that the characteristics of iron oxide NPs, such as their shape, size, surface charge, concentration, functional groups, and type of coating, had an impact on their toxicity (15). Nanomaterials are produced by different methods, like the sol-gel method, co-precipitation, chemical reduction method, and hydrothermal synthesis (16). The chemicals used in these methods have harmful effects on the environment. Thus, green synthesis of nanomaterials has recently attracted interest due to its simplicity, low cost, and environmentally friendly nature (17). Recent studies have described the green synthesis of iron oxide nanoparticles (FeO-NPs) using a variety of biological sources, such as plants (18), seeds (19), and bacteria (20). In the green synthesis of nanoparticles, the plant extract is used as a reducing and capping agent, instead of the toxic reducing chemicals (21). Iron oxide nanoparticles synthesized by curcumin as a reducing agent showed antidepressant activity (22). The authors have shown that curcumin-coated iron oxide nanoparticles exhibit antioxidant effects and increase monoamine neurotransmitters. Moreover, researchers reported that Fe_3_O_4_ magnetic-CurNPs exhibited potent antineurotoxicity activity in the cerebellum of schizophrenic rats (23). 

The present study was performed to evaluate the protective effect of iron oxide (FeO) nanoparticles capped with curcumin against motor impairment and neurochemical alterations in the PD rat model induced by reserpine.

## Materials and Methods

***Animals***

Twenty-four adult male Wistar albino rats (32 weeks old) were used in the present investigation. Their weights ranged from 160–180 g at the beginning of the experiment. They were purchased from the animal house of The National Research Centre, Giza, Egypt. Animals were kept in a controlled environment with a regular light/dark cycle (12/12) and temperature (25 ± 2 °C). Animals were given access to standard food pellets and tap water throughout the experiment *ad libitum*. Animal procedures were performed after the approval of the Institutional Animal Care and Use Committee (IACUC) of Cairo University, Faculty of Science (Egypt) (CU/I/F/25/19).

***Preparation and characterization of FeO NPs-Cur***

The preparation and characterization of iron oxide nanoparticles capped with curcumin were based on the methods reported by Khadrawy *et al*. (22) and Elbialy *et al*. (24).

A mixture of 1 g of FeC_l2_.4H_2_O and 2.5 g of FeC_l3_.6H_2_O were dissolved in 50 ml of deionized water under a constant flow of nitrogen at 40 °C for 15 min. Separately, 2 mg of curcumin (Cur) was dissolved in dimethyl sulfoxide (DMSO) (500 μl) and added dropwise to the mixture, and the temperature was elevated to 85 °C. Five milliliters of 28% ammonium hydroxide were added and the mixture was stirred vigorously for 1 hr. The solution was then washed with deionized water several times, dried at 60 °C, and stored until use.

The prepared formula of curcumin-FeO NPs was imaged by transmission electron microscopes (TEM) (average size of the nanoparticles was 15 ± 3 nm) and the stability of the prepared formula was determined by measuring its zeta potential (-25.4 mV). In addition, FeONPs-Cur were analyzed in the wavelength range of 200 to 700 nm using UV-Vis absorption spectroscopy using a Jenway UV-Vis spectrophotometer (UV-6420, UK). The range of the absorption peaks suggests that FeONPs-Cur are dispersed uniformly in the aqueous solution.

The dose of FeONPs-Cur (5 mg/kg) was selected according to the previous studies of Khadrawy *et al*. (22) and Elbialy *et al*. (24).

***Experimental design***

The twenty-four rats were divided into three groups as follows: 

1-The first group was the control group (n=8) in which rats were injected intraperitoneally with saline (0.9 %) daily for 21 days. On the 22^nd^ day, control rats received two saline injections daily for 14 days. 

2- The second group was the rat model of PD (n=8) induced by the intraperitoneal (IP) injection of reserpine (0.2 mg/kg) daily for 21 days (25). Starting from the 22^nd^ day, the rat model of PD received an IP injection of reserpine (0.1 mg/kg) followed by saline daily for 14 days to maintain the animal model.

3- The third group was the rat model of PD (n=8) induced by the IP injection of reserpine (0.2 mg/kg) daily for 21 days. Starting from the 22^nd^ day, the rat model was treated daily with reserpine (0.1 mg/kg) followed by an IP injection of FeONPs-Cur (5 mg/kg) with one-hour intervals in-between for 14 days. At the end of the experimental period, the rats were tested by the open field test (OFT) to evaluate their motor activity. Then rats were sacrificed and the brain of each rat was removed and divided longitudinally into two equal halves. The striatum and midbrain of each half were dissected out. Then each brain region was weighed and frozen until analysis. The right halves of each of the selected brain regions were used to measure monoamine neurotransmitters (dopamine, norepinephrine, and serotonin). The left halves of the midbrain or striatum were homogenized in Tris-HCl buffer (pH 7.4) and centrifuged at 5000 rpm, 4 °C for 10 min. The rest of the neurochemical parameters including lipid peroxidation, nitric oxide, and reduced glutathione levels together with acetylcholinesterase, monoamine oxidase, and Na^+^,K^+^,ATPase activities were analyzed in the supernatant.

***Open field test (OFT)***

Open field test (OFT) was used to measure motor activity (26) and was made of white plywood that measured 72 x 72 cm with 36 cm walls with one wall of clear plexiglass to allow the observation of the rats. The clear Plexiglas floor of the open field was divided into sixteen squares (18 x 18 cm) with clear blue lines and a central square (18 cm x 18 cm) in the middle. Each rat was placed for ten min in the apparatus. The quantified motor parameters of OFT include central square duration (the duration of time the rats spend in the central square), line crossings (the number of times they cross a grid line with all four paws), rearing (the number of times the rats stand on their hind legs in the maze), and freezing time (the amount of time they remain completely motionless). After each session, the open field device was cleaned with 70% ethyl alcohol and left to dry.

***Neurochemical measurements***

The following parameters were measured in the left half of each midbrain (n=24) and striatum (n=24) of each rat. 

***Lipid peroxidation level (MDA)***

MDA was determined in the midbrain and striatum by measuring the level of malondialdehyde, one of the main lipid peroxide metabolites. The procedure was based on the method of Ruiz-Larrea *et al*. (27) in which malondialdehyde interacts with thiobarbituric acid in an acidic medium yielding a pink-colored derivative whose absorbance was read at 532 nm using a UV-visible spectrophotometer.

***Nitric oxide level (NO)***

NO level was measured in the midbrain and striatum as the nitrite level using Griess reagent. This reagent reacts with nitrite to form a deep purple-colored derivative whose absorbance was read at 540 nm (28).

***Reduced glutathione level (GSH)***

GSH was determined spectrophotometrically in the midbrain and striatum. In this method, 5,5` dithiobis (2 - nitrobenzoic acid) was reduced with glutathione (GSH) to produce a yellow compound whose absorbance was measured at 405 nm (29). The reduced chromogen was directly proportional to the GSH level.

***Acetylcholinesterase activity (AchE)***

AchE activity was determined by the method of Gorun *et al*. (30) in which acetylthiocholine iodide was hydrolyzed by the enzyme to yield thiocholine. DTNB is reduced to thionitrobenzoic acid after the reaction of its –SH groups with thiocholine. The yellow color of thionitrobenzoic acid was read spectrophotometrically at 412 nm. 

***Na*** ^+^ ***,K*** ^+^ ***ATPase activity***

Na^+^,K^+^-ATPase activity was measured by the spectrophotometric method described by Tsakiris *et al*. (31). Mg^2+^-dependent ATPase activity was subtracted from total ATPase activity (Na^+^,K^+^,Mg^2+^-dependent) to give Na^+^,K^+^-ATPase activity. The color developed was read at 640 nm.

***Monoamine oxidase activity (MAO)***

MAO activity was measured by the method of Holt *et al*. (32) in which benzylamine is converted to benzaldehyde whose absorbance was measured at 250 nm.

***Monoamine levels***

The determination of serotonin (5-HT), norepinephrine (NE), and dopamine (DA) was based on the method of Ciarlone (33). Homogenization of the right half of each midbrain and striatum was carried out in acidified butanol followed by centrifugation at 3000 rpm for 5 min. In a test tube, 2.8 ml of the supernatant was added to 1.6 ml acetic acid (0.2 N) and 5 ml heptane. Then the tubes were vortexed for about 30 sec and centrifuged again at 3000 rpm for 5 min. The organic layer was removed, and the aqueous layer was analyzed to measure the monoamines using a spectrofluorometer (Jasco FP-6500, JASCO Ltd., Tokyo, Japan) equipped with a xenon arc lamp 150 W (excitation and emission slit bandwidth of the excitation and emission monochromator was 5 nm).

***Statistical analysis***

All results were presented as mean ± SEM. Data were analyzed by one-way analysis of variance (ANOVA) followed by Duncan as *post hoc* test to calculate the difference between groups. The significance level was set at *P-value*<0.05. 

## Results

***Open field test***

**Figure 1:**
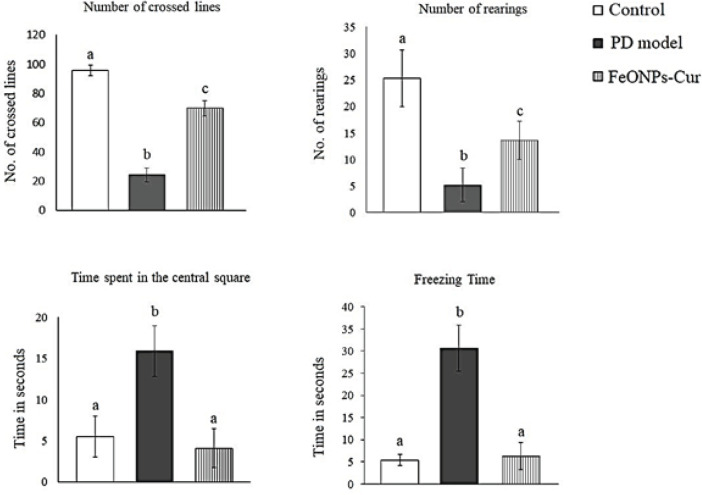
Effect of daily treatment with FeONPs-Cur (5 mg/kg/day for 14 days) on the open field test parameters (Number of crossed lines, Number of rearings Time spent in the central square, and Freezing time) in the reserpine-induced PD rat model

One-way analysis of variance (ANOVA) test revealed that the mean time spent by the rat model of PD in the central square (15.9 ± 1.09 sec) and the freezing time (30.6 ± 1.83 sec) increased significantly (*P*=0.001) recording 189.1% and 466.6%, respectively, compared to those of the control rats (5.5 ± 0.88 and 5.4 ± 0.46 sec). In addition, the number of crossed lines (24.3 ± 1.13) and the number of rearings (5.2 ± 1.13) performed by the rat model of PD decreased significantly (*P*=0.001) compared to the control values (95.7 ± 1.20 and 25.3 ± 1.91, respectively) (Figure 1).

When the PD rat model was treated with FeONPs-Cur, the time spent by treated rats in the central square (4.1 ± 0.85) and the freezing time (6.3 ± 1.09) showed non-significant changes compared to control rats. FeONPs-Cur treatment also elevated the number of crossed lines (83.7 ± 1.27) and the number of rearings (15.8 ± 1.23), however, they did not return to the control values (Figure 1). 

***Oxidative stress***

**Figure 2:**
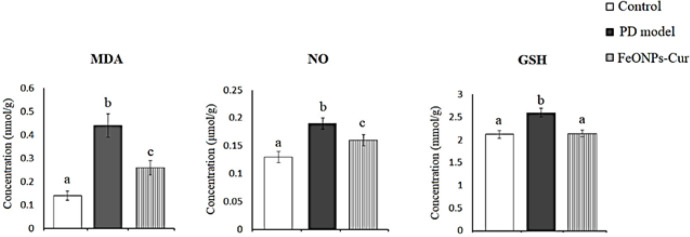
Effect of daily treatment with FeONPs-Cur (5 mg/kg/day for 14 days) on the level of lipid peroxidation (MDA)(mmol/g), nitric oxide (NO) (µmol/g), and reduced glutathione (GSH) (mmol/g) in the midbrain of reserpine-induced PD rat model

ANOVA test revealed in the midbrain of the rat model of PD a significant increase in the levels of lipid peroxidation (MDA) (0.44 ± 0.05, *P*=0.001), nitric oxide (NO) (0.13 ± 0.01, *P*=0.001), and reduced glutathione (GSH) (2.60 ± 0.09, *P*=0.001) recording +214.3%, +46.15%, and +22.64%, respectively, were observed compared to control values (0.14 ± 0.02, 0.13 ± 0.01, and 2.12 ± 0.08, respectively). Although the daily treatment with FeONPs-Cur reduced the increase in the levels of MDA and NO obtained in the rat model of PD to +85.71% and +23.08%, respectively, MDA (0.26 ± 0.03, *P*=0.001) and NO (0.16 ± 0.01, *P*=0.001) did not return to control values. However, GSH (2.14 ± 0.07, *P*>0.05) showed a control-like value after FeO NPs-Cur treatment (Figure 2).

**Figure 3:**
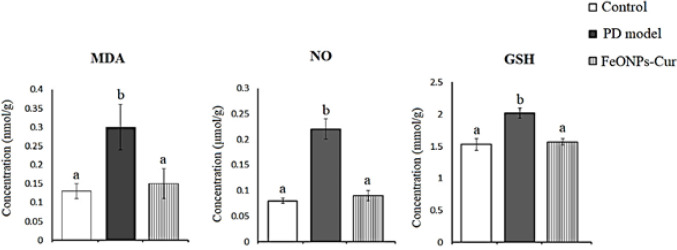
Effect of daily treatment with FeONPs-Cur (5 mg/kg/day for 14 days) on the level of lipid peroxidation (MDA)(mmol/g), nitric oxide (NO) (µmol/g)), and reduced glutathione (GSH) (mmol/g) in the striatum of reserpine-induced PD rat model

Similarly, in the striatum of the rat model of PD, MDA (0.30 ± 0.06, *P*=0.008), NO (0.22 ± 0.02, *P*=0.001) and GSH (2.02 ± 0.08, *P*=0.001) increased significantly recording +130.77%, +175%, and +23.03% as compared to control values (0.13 ± 0.02, 0.08 ± 0.005 and 1.53 ± 0.09), respectively. FeONPs-Cur restored MDA (0.14 ± 0.01), NO (0.09 ± 0.01) and GSH (1.57 ± 0.05) to control-like values (Figure 3).

***Na*** ^+^ ***,K*** ^+^ ***,ATPase, acetylcholinesterase, and monoamine oxidase***

**Figure 4:**
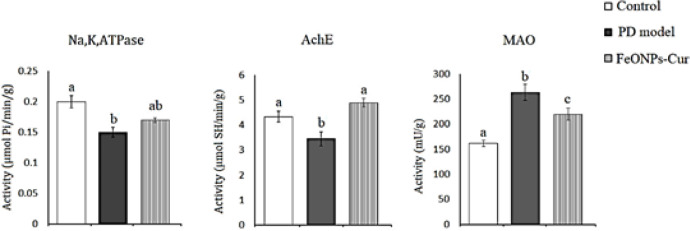
Effect of daily treatment with FeONPs-Cur (5 mg/kg/day for 14 days) on the activity of Na+,K+,ATPase (µmol Pi/min/g), acetylcholinesterase (AchE) (µmol SH/min/g))), and monoamine oxidase (MAO) (mU/g) in the midbrain of reserpine-induced PD rat model

In the midbrain of the rat model of PD, ANOVA revealed a significant decrease in the activity of Na^+^,K^+^,ATPase (0.15 ± 0.008, *P*=0.026) and acetylcholinesterase (AchE) (3.45 ± 0.28, *P*=0.002) by 25% and 20.51% compared to control rats (0.20 ± 0.01 and 4.34 ± 0.22, respectively). However, the activity of monoamine oxidase (MAO) (263.61 ± 15.59, *P*=0.001) increased significantly by 34.56% compared to the control value (161.64 ± 5.96). FeONPs-Cur treatment improved the activity of Na^+^,K^+^,ATPase (0.17 ± 0.005) to -15% recording nonsignificant changes as compared to the control and rat model of PD. FeONPs-Cur treatment restored AchE (4.90 ± 0.17) activity to control-like value and reduced the increased MAO activity (219.81 ± 11.89, *P*=0.001) induced in the midbrain of the rat model of PD to be significantly changed as compared to control and rat model of PD (Figure 4).

**Figure 5:**
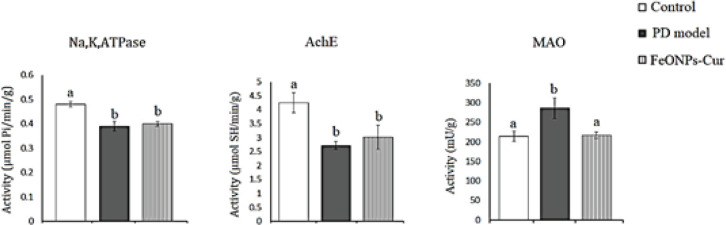
Effect of daily treatment with FeONPs-Cur (5 mg/kg/day for 14 days) on the activity of Na+, K+, ATPase (µmol Pi/min/g), acetylcholinesterase (AchE) (µmol SH/min/g), and monoamine oxidase (MAO) (mU/g) in the striatum of reserpine-induced PD rat model

In the striatum of the rat model of PD, ANOVA revealed that the activity of Na^+^,K^+^,ATPase ( 0.39 ± 0.02, *P*=0.001) and AchE (2.71 ± 0.14, *P*=0.001) decreased significantly by 18.75% and 36.24% as compared to control values (0.48 ± 0.01, 4.25 ± 0.036), respectively. However, a significant increase was observed in the MAO activity (286.32 ± 26.06, *P*=0.009) recording +33.40% compared to control values (214.63 ± 13.22). FeONPs-Cur failed to ameliorate the decreased activity of Na^+^,K^+^,ATPase (0.40 ± 0.01) and AchE (3.01 ± 0.44) induced in the rat model of PD but restored MAO (209.79 ± 2.37, *P*>0.05) to control-like activity (Figure 5).

***Monoamine neurotransmitters***

**Figure 6:**
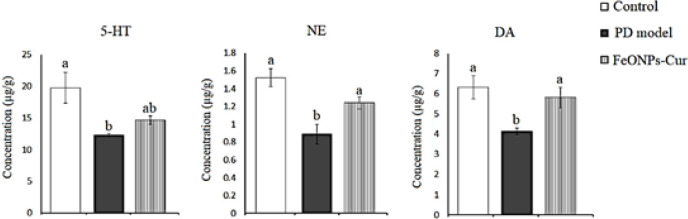
Effect of daily treatment with FeONPs-Cur (5 mg/kg/day for 14 days) on the level of serotonin (5-HT) (µg/g), norepinephrine (NE) (µg/g)), and dopamine (DA) (µg/g) in the midbrain of reserpine-induced PD rat model

In the midbrain of the rat model of PD, ANOVA revealed a significant decrease in serotonin (5-HT) (12.29 ± 0.23, *P*=0.019), norepinephrine (NE) (0.89 ± 0.11, *P*=0.001), and dopamine (DA) (4.15 ± 0.16, P = 0.14) in the midbrain of the rat model of PD recording -37.71%, -41.45% and -34.34%, respectively, as compared to control values (19.73 ± 2.43, 1.52 ± 0.10, and 6.32 ± 0.58, respectively). FeONPs-Cur treatment ameliorated the deceased level of NE (1.24 ± 0.07) and DA (5.81 ± 0.52) recording non-significant changes as compared to control values. Although the reduced level of 5-HT (14.62 ± 0.68) was improved by FeONPs to no significant change as compared to the control value, it still showed no significant change compared to the PD model (Figure 6). 

**Figure 7:**
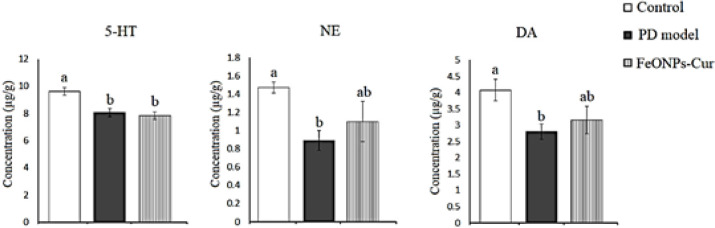
Effect of daily treatment with FeONPs-Cur (5 mg/kg/day for 14 days) on the level of serotonin (5-HT) (µg/g), norepinephrine (NE) (µg/g)), and dopamine (DA) (µg/g) in the striatum of reserpine-induced PD rat model

In the striatum of the rat model of PD, 5-HT (8.06 ± 0.31, *P*=0.001), NE (0.89 ± 0.11, *P*=0.024), and DA (2.80 ± 0.23, *P*=0.038) decreased significantly by 16.13%, 39.46%, and 31.20%, respectively, as compared to control values (9.61 ± 0.29, 1.47 ± 0.06, and 4.07 ± 0.33, respectively). Although FeONPs-Cur elevated the reduced level of NE (1.10 ± 0.22) and DA (3.16 ± 0.42) recording non-significant changes as compared to the control and rat model of PD, the decreased 5-HT (7.84 ± 0.28, *P*=0.001) level did not show any improvement (Figure 7).

## Discussion

The present data revealed that Fe2O3NPs-Cur improved the behavioral and neurochemical changes in the midbrain and striatum of rat models of PD induced by reserpine. This was clear from its ability to prevent the declined DA, NE, and 5-HT. In addition, Fe2O3NPs-Cur ameliorated oxidative stress and the changes in the activities of MAO, AchE and Na^+^,K^+^,ATPase 

The present rat model of PD induced by reserpine is a successful model that is used in the screening of antiparkinsonian agents (25). This model is characterized by motor (34) and non-motor symptoms (35) like those recorded in humans. The appearance of such symptoms in rats is attributed to the depleting effect of reserpine on monoamine neurotransmitters.

These features occur as a consequence of the blockage of vesicular monoamine transporter-2 (VMAT2) by reserpine (36), resulting in monoamine depletion, including DA, NE, and 5-HT. This may explain the reduced monoamine levels in the midbrain and striatum in the present study. The blockage of VMAT2 by reserpine prevents the influx of free cytoplasmic monoamines into their vesicles exposing them to their metabolizing enzyme, monoamine oxidase, after their reuptake from the synaptic cleft into the presynaptic monoaminergic nerves. Therefore, the increased MAO activity in the midbrain and striatum of the present PD model could be a result of the blockage of VMAT2 to metabolize the extravesicular monoamine neurotransmitters.

One of the main insults of the increased MAO activity is the increased production of oxidative stress that is implicated in the pathophysiology of PD (5, 37). Thus, the oxidative stress induced by reserpine may be related to the increased dopamine metabolism which results in reduction in DA molecules in the vesicles and increased DA turnover.

The present data also revealed an increase in lipid peroxidation (MDA) and nitric oxide (NO) in the midbrain and striatum of the rat model of PD, confirming the production of oxidative stress in the two studied brain regions. The increased lipid peroxidation is caused by the attack of the cell membrane by the increased free radicals, resulting in phospholipid oxidation and cell death. Researchers have found that the production of free radicals stimulates lipid peroxidation and cell death in neurons and astrocytes, where free radicals also attack DNA and protein, causing cellular damage and death (38). The increased GSH levels in the striatum and midbrain could be a compensatory feedback mechanism to combat the production of free radicals.

 Post-mortem studies revealed increased expression of neuronal nitric oxide synthase (nNOS) in the midbrain samples of PD patients (39) and animal models of PD (40). Similarly, nNOS is increased in the substantia nigra of weaver mice, characterized by the spontaneous depletion of dopaminergic neurons (41). Therefore, the present increase in NO in the midbrain and striatum may be due to the increased expression and activity of nNOS. NO may interact with free oxygen, forming the damaging molecule peroxynitrite, which causes protein and DNA nitrosylation. 

PD mainly influences the motor system by degenerating the dopaminergic neurons, which lose their ability to produce dopamine, eventually leading to damage to the motor cortex and movement disorders (3). Motor control and sensory processing are governed by dopamine (42) and norepinephrine (43), which coordinate the optimized behavior.

The open-field test parameters measured in the present study indicate impairment in motor activity in the rat model of PD. This was indicated by the significant increase in the freezing time and the time maintained by rats in the central square and the decrease in the number of crossed lines and the number of rearings. The present decrease in motor activity could be attributed to the decreased levels of dopamine and norepinephrine which mediate the impaired motor activity induced by reserpine in the current PD model.

Postmortem, animal, and functional imaging studies (44, 45) have shown the involvement of serotonergic dysfunction in PD. The present decrease in serotonin levels in the midbrain and striatum of the PD model confirms its role in the pathogenesis of PD. This decrease is postulated to modulate different cognitive and physiological deficits in mood, sleep, emotion, and appetite; thus, variations in serotonergic neurotransmission may contribute to the nonmotor disturbances observed in PD (46).

Although the fundamental neurochemical change in PD is the depletion of dopamine, deficits in cholinergic transmission have also been observed in PD patients (47). Midbrain DA neurons are modulated by the endogenous cholinergic system, that originates in the mesopontine nuclei. Dopaminergic neurons in the substantia nigra pars compacta and the ventral tegmental area are supplied by dense cholinergic innervation from mesopontine cholinergic nuclei, including the pedunculopontine tegmental nucleus (PPN) and the laterodorsal tegmental nucleus (48). When the PPN neurons are stimulated, the firing rate of DA neurons increases in the ipsilateral substantia nigra by nicotinic cholinergic receptor activation (48) indicating the stimulating effect of these cholinergic nuclei on midbrain DA neurons. Therefore, the reduced AchE activity induced by reserpine in the midbrain of the present PD model could be attributed to the cholinergic neuron degeneration that has been reported by Jellinger in the PPN nucleus in PD (49). Using [^11^C] PMP PET, a significant inhibition in thalamic AchE activity, which mainly stems from PPN projections, has been recorded in PD patients (50). 

Cholinergic interneurons in the striatum play an important role in the normal functioning of the basal ganglia in movement control (51). The interplay between DA and Ach in the striatum lends support to the idea that the cholinergic and dopaminergic systems cause movement through opposing activities and that pathological states are characterized by imbalanced interactions (52).  The depletion of dopamine in PD prevents muscarinic autoreceptors from autoinhibiting acetylcholine release, leading to an excessive neurotransmitter release; this prunes the spines of the striatal projection neurons of the indirect pathway and thus interferes with the transfer of information coming from motor command centers in the cerebral cortex (52). Thus, the present reduction of AchE induced by reserpine in the striatum exacerbates the hypercholinergic activity and consequently the motor deficits. Furthermore, the oxidative stress observed in the midbrain and striatum in the current rat model of PD may play a role in reducing AchE activity (31). 

In the present study reduced Na^+^,K^+^,ATPase activity was observed in the midbrain and striatum of the rat model of PD as compared to control rats. Na^+^,K^+^,ATPase dysfunction has been observed in PD rodent models and patients [53]. Furthermore, restoring Na^+^,K^+^,ATPase activity and membrane stability in neurons alleviated dopaminergic neurodegeneration. These results indicate a strong correlation between Na^+^,K^+^,ATPase and the pathogenesis of PD. The decrease in Na^+^,K^+^,ATPase activity in the striatum and midbrain could also be caused by free radicals produced by the oxidative catabolism of monoamines induced by monoamine oxidase. 

Furthermore, decreased Na^+^,K^+^,ATPase may be one of the factors contributing to dopaminergic neuron damage. The pump maintains Na^+^/K^+^ balance across cell membranes and is necessary for proper cell functions (54). Dysfunctional Na^+^/K^+^ pump has been shown to induce neuronal death (55). 

In the present study, iron oxide nanoparticles were prepared by a green method using curcumin acting as a reducing and stabilizing agent (56). By this method, FeONPs capped with curcumin were obtained. The zeta potential of FeONPs capped with curcumin was -25.4 mV which predisposes high stability for the prepared formula (22). This value of zeta potential indicates the absence of nanoparticle agglomeration and the attenuated propensity of nanoparticles to interact with blood plasma proteins (57). Curcumin has many therapeutic effects, but its low bioavailability reduces its therapeutic efficiency. By this method, curcumin gains its nanoform which thereafter increases its bioavailability and decreases the toxic effects that may be induced by iron oxide. 

Researchers have demonstrated that FeONPs-Cur has antidepressant activity (22). The study revealed that FeONPs-Cur increased monoamine levels and ameliorated the oxidative stress obtained in the cortex and hippocampus of the rat model of depression.

Treatment of the PD rat model with FeONPs-Cur improved motor activity. This was indicated by OFT parameters, which showed control-like values in the time spent by rats in the central square and the freezing time, and an improvement in the number of crossed lines and rearings. This improvement could be caused by the ability of FeONPs-Cur to prevent the decline in DA and NE levels in the midbrain and striatum. This effect may be ascribed to the control-like activity of MAO obtained in rats treated with FeONPs-Cur, which prevented the increased metabolism of catecholamines.

On the other hand, iron plays an important role in neuronal metabolism because it is involved in the synthesis and packaging of neurotransmitters (58). Moreover, iron is a cofactor for tyrosine hydroxylase, a key rate-limiting enzyme responsible for the synthesis of monoamines (59). This may explain the ability of FeNO-Cur to elevate the levels of DA and NE and prevent their decline in the midbrain and striatum of the rat model of PD. 

The present findings showed that FeONPs-Cur improved the 5-HT level in the midbrain. However, in the striatum, FeONPs-Cur failed to induce such improvement. This partial improvement in the level of 5-HT may be responsible for the incomplete recovery in motor activity. Despite an increase in the number of crossed lines and rearings, their levels remained lower than the control values. 

In the midbrain, although FeONPs-Cur reduced the elevated levels of MDA and NO and the increased MAO activity induced by reserpine, their levels were not completely restored to control values but still showed a significant increase. The increase in the oxidative stress parameters may be attributed to the oxidative metabolism of monoamines by the increased MAO activity. However, in the striatum, the elevated levels of DA and NE were associated with a control-like activity of MAO. This may explain the restored levels of MDA, NO, and GSH in the striatum of rats treated with FeONPs-Cur.

The ability of FeONPs-Cur to attenuate the oxidative stress in the striatum could also be exerted by the antioxidant activity of FeONPs, which has catalase-like activity (60). Fe_3_O_4_ NPs exhibit pH-dependent enzyme-like activities. In an acidic solution, Fe_3_O_4_ NPs display peroxidase-like activity, breaking down H_2_O_2_ to produce the hydroxyl radical (OH), but in a neutral solution, they exhibit catalase-like activity which decomposes H_2_O_2_ into H_2_O and O_2_ (60). In addition, the curcumin coat surrounding the FeONPs may contribute to the antioxidant activity induced by the present formula. Several studies have recorded the powerful antioxidant activity of curcumin.

The study by Lakshmi et al. showed that iron oxide nanoparticles synthesized by the use of the aqueous extract of *Convolvulus pluricaulis* had positive memory-improving effects because of their antioxidant property at all doses, but the highest dose had more potency than the standard drug Donepezil against cognitive impairment induced by scopolamine in mice (61).

The current data also revealed that the midbrain AchE activity was restored to a control-like level. This could be attributed to the restored DA level by FeONPs-Cur where Ach has a stimulatory effect on the dopaminergic neurons in the midbrain. However, the inability of FeONPs-Cur to restore striatal AchE activity may be due to the incomplete recovery in the DA level. Similarly, FeONPs-Cur improved Na^+^,K^+^,ATPase activity in the midbrain but had no effect in the striatum.

ANOVA followed by the Duncan *post hoc* test was used to determine the significance between control, PD model, and PD model treated with FeONPs-Cur. Data are expressed as mean± SEM (n=8). Statistically significant means (*P*-value<0.05) are given different letters and the same letters indicate nonsignificant changes.

ANOVA followed by the Duncan post hoc test was used to determine the significance between control, PD model, and PD model treated with FeONPs-Cur. Data are expressed as mean± SEM (n=8). Statistically significant means (*P*-value<0.05) are given different letters and the same letters indicate nonsignificant changes.

ANOVA followed by the Duncan *post hoc *test was used to determine the significance between control, PD model, and PD model treated with FeONPs-Cur. Data are expressed as mean± SEM (n=8). Statistically significant means (*P*-value<0.05) are given different letters and the same letters indicate nonsignificant changes.

ANOVA followed by the Duncan *post hoc *test was used to determine the significance between control, PD model, and PD model treated with FeONPs-Cur. Data are expressed as mean± SEM (n=8). Statistically significant means (*P*-value<0.05) are given different letters and the same letters indicate nonsignificant changes.

ANOVA followed by the Duncan *post hoc* test was used to determine the significance between control, PD model, and PD model treated with FeONPs-Cur. Data are expressed as mean± SEM (n=8). Statistically significant means (*P*-value<0.05) are given different letters and the same letters indicate nonsignificant changes.

ANOVA followed by the Duncan *post hoc* test was used to determine the significance between control, PD model, and PD model treated with FeONPs-Cur. Data are expressed as mean± SEM (n=8). Statistically significant means (*P*-value<0.05) are given different letters and the same letters indicate nonsignificant changes

ANOVA followed by the Duncan *post hoc* test was used to determine the significance between control, PD model, and PD model treated with FeONPs-Cur. Data are expressed as mean± SEM (n=8). Statistically significant means (*P*-value<0.05) are given different letters and the same letters indicate nonsignificant changes

## Conclusion

It is clear from the present data that FeONPs-Cur ameliorated the motor impairment induced in the PD rat model. This could be mediated by the elevated level of dopamine in the midbrain and striatum. Moreover, the improved norepinephrine level in the two brain regions may contribute to reducing the motor and nonmotor complications in the rat model of PD induced by reserpine. In addition, the antioxidant activity exerted by FeONPs-Cur could also potentiate its neuroprotective effects against the rat model of PD.

In light of the present findings, FeONPs-Cur could be recommended as promising adjuvant therapy against PD.

## Authors’ Contributions

Y AK and H M conceived the study. H M synthesized and characterized FeONPs-Cur. Y AK and M HS performed the behavioral test. Y AK and E H carried out the biochemical analyses. E H performed the statistical analyses. Y AK wrote the manuscript. All authors reviewed and approved the manuscript. 

## Recommendations

Further studies are needed to evaluate FeONPs-Cur in other animal models of PD elaborating the behavioral tests and measured parameters to confirm the antiparkinsonian effect of FeONPs-Cur. 

## Funding

No funding was obtained from any agency.

## Data Availability

Data will be made available upon request.

## Conflicts of Interest

None.
